# Magneto-Mechanical Coupling Modeling and Full-Cycle Characterization of V-Shaped Crack Evolution in Q345 Steel Using Metal Magnetic Memory

**DOI:** 10.3390/ma19101980

**Published:** 2026-05-11

**Authors:** Cheng Xu, Haiyan Xing, Liwei Zhao, Haibo Miu, Hai Zhang

**Affiliations:** 1 School of Mechanical and Electronic Engineering, Qiqihar University, Qiqihar 161006, China; 02649@qqhru.edu.cn; 2 School of Mechanical Science and Engineering, Northeast Petroleum University, Daqing 163318, China; 241971020097@nepu.edu.cn; 3 Wenzhou Special Equipment Inspection and Research Institute, Wenzhou 325009, China; tjlwzhao@163.com (L.Z.); zjwzmhb@126.com (H.M.)

**Keywords:** metal magnetic memory, crack evolution, magneto-mechanical coupling, magnetic dipole model, Q345 steel

## Abstract

Metal magnetic memory (MMM) is a promising non-destructive evaluation method for ferromagnetic materials, allowing early detection of stress concentration and micro-defects under weak geomagnetic excitation. However, current magneto-mechanical coupling models are computationally complex and insufficient to characterize the full-cycle evolution of mesoscale physically short cracks. This work proposes a magnetic dipole model and its decomposed formulation for V-shaped cracks. Combined with theoretical derivation, finite element simulation, and in situ three-point bending tests on Q345 steel, the magneto-mechanical coupling mechanism and magnetic signal evolution during crack propagation are investigated. Results show that the MMM normal component exhibits obvious peak-peak features at the crack tip, while the tangential component shows a single-peak characteristic. Two critical signal mutations are observed at crack lengths of about 100 μm and 3000 μm, corresponding to micro-meso and meso-macro crack transitions, respectively. The model is verified with relative errors of 15.2% for *H_x_* and 17.6% for *H_y_*. This study reveals the quantitative correlation between MMM signals and full-lifecycle crack growth, supporting damage assessment and fatigue life prediction for ferromagnetic engineering structures.

## 1. Introduction

Ferromagnetic materials are widely employed in various industrial structures and core equipment in fields such as mechanical manufacturing, pipeline transportation, and aerospace. During service, these materials are susceptible to loading, corrosion, fatigue, and other influencing factors, leading to the initiation of surface and internal crack defects. The continuous propagation of cracks can result in structural failure, triggering severe safety accidents and economic losses [1]. Conventional non-destructive testing (NDT) techniques, including X-ray testing, ultrasonic testing, and magnetic flux leakage testing, enable the detection of macroscopic cracks. However, they have limited ability to identify early-stage hidden cracks and subsurface cracks. Additionally, some techniques require preprocessing of the testing surface, involving complex operational procedures that fail to meet the detection requirements under complex working conditions [2,3,4].

MMM technology is grounded in the magnetoelastic effect of ferromagnetic materials. Under the coupled effect of the natural geomagnetic field and external stress, magnetic domain reconstruction occurs in crack and stress concentration regions, generating characteristic magnetic flux leakage signals. By detecting and analyzing these signals, the localization, characterization, and evaluation of cracks can be achieved [5]. Owing to its unique advantages such as no need for external excitation, non-contact operation, and online detectability, this technology has become a research hotspot in the field of crack detection [6]. In recent years, scholars have conducted extensive research on the theoretical mechanism, crack characterization methods, evaluation criteria, and engineering applications of MMM technology, yielding abundant research outcomes.

Regarding the fundamental theoretical research of MMM technology for crack detection, Leng [7] adopted a linear magnetic dipole model to investigate the dislocation density distribution in the plastic zone at the tip of V-shaped cracks. Liu [8] utilized rectangular grooves with varying widths and depths to simulate surface cracks, exploring the influence of crack size on stress evaluation via magnetic memory technology. Jung [9] identified significant differences in magnetic field intensity between cracked and uncracked regions, verifying the feasibility of MMM technology for subsurface defect detection. Lopez-Huerta [10] achieved the detection of local magnetic distortion around cracks in metallic materials through numerical simulation of the magnetic field around rectangular defects. Bao [11] performed tensile tests on diamond-shaped crack specimens and found that cracks alter the signal distribution characteristics and distortion degree but do not affect the overall signal pattern. Li [12] proposed a double rectangular crack hypothesis for three-dimensional magnetic signal problems and established a theoretical analysis model of magnetoelastic coupled magnetic dipoles. It was discovered that the MMM signal exhibits a superposition effect of magnetic signals from two cracks when the spacing between them is small, refining the MMM theoretical model for multi-crack scenarios.

In the study of MMM characterization of crack size, Huang [13] observed an approximately linear relationship between the maximum gradient of the normal component of the magnetic signal and crack length during the mid-term of crack propagation. The normal component of the signal at the crack center is consistently greater than that at the crack tip. Li [14] identified linear correlations between two characteristic parameters of MMM—the peak value of the tangential component and the maximum gradient value of the normal component—and fatigue crack length. Fan [15,16] carried out four-point bending tests and confirmed good linear correlations between crack length and the minimum value of the tangential component of MMM signals, as well as the maximum values of normal and tangential gradients. Moreover, the signal distribution characteristics are closely associated with the cumulative degree of plastic deformation at the crack tip. Li and Su [17] investigated the variation laws of signal curves and gradient defects with crack length at equal-thickness butt welds, finding that signal curves show abrupt fluctuations while the maximum gradient remains stable, with the errors between predicted and tested crack lengths within 16%. Ye [18] identified linear correlations between the gradient values of magnetic memory signals and both the stress intensity factor and crack length during crack propagation in Q235 steel. Ni [19] discovered a linear correlation between the maximum gradient of MMM signals and crack depth. Xu [20] demonstrated that crack depth, external applied load, heat treatment process, and other factors exert significant influences on magnetic memory signal characteristics, and further revealed that the magnetic memory gradient can characterize the propagation of buried welding cracks. Lee [21] investigated three magnetic models for estimating fatigue crack depth, and the established improved magnetic dipole model enables the evaluation of crack propagation. Bao [22] measured the Residual Magnetic Field (RMF) signals on the surface of arc additive manufacturing (WAAM) steel specimens containing cracks, finding that characteristic parameters are related to crack width. Liu [23] observed that the axial component of pipeline welding cracks exhibits a unimodal distribution, while the radial component presents two pairs of zero-crossing peaks—the outer peaks correspond to the welding zone and the inner peaks correspond to the crack zone. The signal characteristic values show a linear positive correlation with crack width and internal pipeline pressure.

In research on crack evaluation criteria and inversion, Su [24,25,26] established a stress ratio-dependent model, clarifying that the abrupt peak-valley values and their gradients of MMM signals can effectively characterize crack locations, providing explicit criteria for crack positioning evaluation. Deng [27] found that when the crack width is much larger than the lift-off value of the magnetic field sensor, the distribution of the defect magnetic flux leakage field becomes more complex, posing greater challenges to image inversion of defects. Li [28] conducted online DIC testing, analyzed the correlation between the longitudinal strain curve of the crack region in plate specimens and MMM detection results, and proposed three novel indicators for measured data analysis. Liang [29] adopted multidimensional data-enhanced evaluation indicators and a CNN classification model to improve the accuracy and precision of multi-class defect recognition, with crack detection precision increased by 8.8%. Shi [30] implemented a conjugate gradient inversion algorithm to achieve quantitative evaluation of stress concentration and surface cracks in ferromagnetic materials. The effectiveness of this method in determining defect location and size was experimentally verified, and the influences of detection parameters on quantitative results were systematically analyzed.

To address the challenges of crack detection in practical engineering structures, Xin [31] proposed an online detection and characterization model for subsea pipeline cracks based on spatial magnetic signal extraction technology and machine learning algorithms. Guo [32] conducted magnetic memory technology research on crack detection for 25Cr35NiNb ethylene cracking furnace tubes, validating the effectiveness of MMM technology via penetration testing and scanning electron microscopy. Anatoly Dubov [33] discussed the application of the MMM method in the internal surface inspection of boiler heat exchanger steel tubes; metallographic studies and tests confirmed that MMM can detect metal damage in the form of microcracks. Villegas-Saucillo [34] measured magnetic memory signals around small V-shaped notches on steel tubes, and the findings of this study can be applied to the real-time monitoring of V-shaped cracks in steel pipelines.

At present, research on magnetic memory technology has been mainly focused on magnetic feature extraction and characterization under dynamic fatigue loading and at the macroscopic scale, while insufficient investigation has been conducted on the full-cycle characterization of cracks, including mesoscale physically short cracks. To reveal the intrinsic magneto-mechanical coupling mechanism during crack evolution, in this paper, a magnetic dipole model and its decomposed formulation are established based on V-shaped cracks, which reduces high computational complexity and improves engineering applicability encountered in crack characterization using MMM. On the basis of in situ observation and synchronized magnetic memory testing experiments, the evolution laws of magnetic signal characteristics during the entire propagation process of physical cracks are analyzed, and a correlation model covering the full life cycle of cracks is constructed. This work is expected to provide a theoretical reference for the characterization and evaluation of crack growth in engineering practice.

## 2. Modeling

As the stress concentration zone is located at the crack tip during crack propagation, the stress field at the crack tip redistributes with continuous crack evolution, and the domain structure at the crack tip also exhibits nonlinear variations accordingly.

Under sustained loading, with crack development, significant plastic slip occurs in the small stress-concentrated region, accompanied by the accumulation of substantial stress energy. During crack propagation, the material properties in the cracked region change, leading to an obvious variation in magnetic permeability. The non-uniform stress around the crack strongly disturbs the local magnetic structure. At the microscopic level, magnetic moment rotation and domain wall motion are impeded, hindering the magnetization process. Under normal conditions, the material can be magnetized in an external magnetic field. However, due to the above obstructions, local magnetization near the crack is insufficient, resulting in non-uniform magnetic distribution and the accumulation of magnetic charges around the crack.

**Figure 1:**
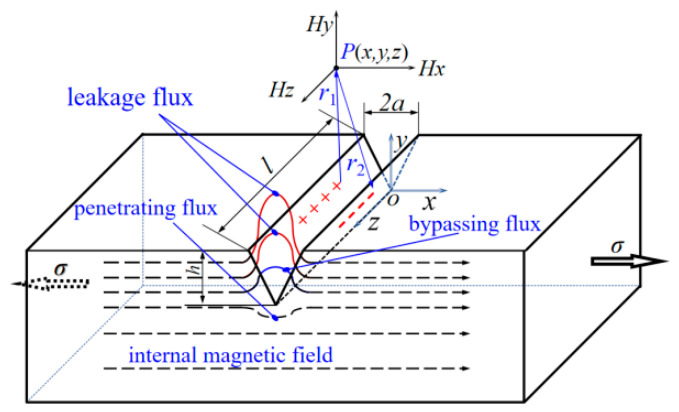
Magnetic Charge Principle of Crack Magnetic Leakage.

Actual surface cracks in metals inherently exhibit a wedge-shaped profile with a wide opening and a gradually tapered tip, which is geometrically consistent with the V-shaped configuration. The V-shaped simplification can well characterize key morphological features, including crack depth, opening angle, and stress concentration effect at the crack tip. Meanwhile, its regular geometric boundary facilitates coordinate transformation, vector decomposition, and integral simplification in magnetic field modeling, which balances physical rationality and mathematical feasibility. Accordingly, this simplification is suitable for the quantitative characterization of cracks in metal magnetic memory detection. A rectangular coordinate system is established as shown in Figure 1, with the origin located at the bottom of the V-shaped crack. The x-direction is parallel to the tensile direction, the y-direction is parallel to the normal direction of the specimen surface, and the z-axis is along the crack length. The crack opening width is denoted as 2*a*, the crack depth as *h*, and the crack length as *l*.

The magnetic medium on both sides of the crack changes, leading to a variation in magnetic permeability within the magnetic circuit. Meanwhile, under magnetic field excitation, the magnetic flux in the ferromagnetic material is distorted and leaked. For ferromagnetic structural components with surface cracks, the degree of plastic deformation and magnetization state around the crack vary gently along the crack propagation direction, resulting in relatively uniform accumulation of magnetic charges on the crack surface [7]. The assumption of uniform surface magnetic charge density effectively simplifies the derivation of magnetic field integrals while retaining the dominant characteristics of magnetic field distribution. The approximation error is mainly confined to the local crack tip region and remains acceptable within the conventional engineering detection scale, rendering this assumption widely adopted in magnetic dipole models for metal magnetic memory research. Assume that the magnetic charges on both side surfaces of the crack are uniformly distributed, where the two surface elements are (*x*_1_, *y*_1_, *z*_1_) and (*x*_2_, *y*_2_, *z*_2_), respectively, and the detection point in the space near the crack is set as P(*x*, *y*, *z*).

**Figure 2:**
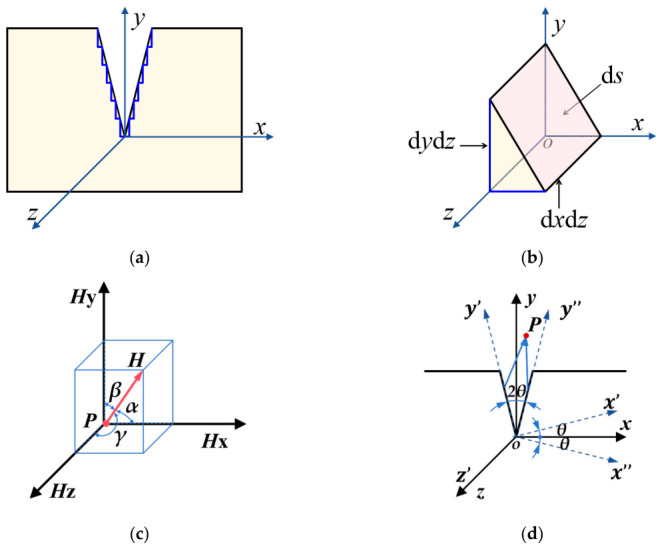
Equivalent Method for Crack Microelements: (**a**) alternative to inclined plane; (**b**) equivalent of the surface element; (**c**) decomposition of magnetic field intensity vector; (**d**) conversion of model coordinates.

Since the surface of an actual crack is not smooth, it is more realistic to model the crack as a stepped shape. Therefore, the inclined surface elements are projected onto the *xoz* plane and *yoz* plane, respectively, as shown in Figure 2a,b.

The magnetic field strength ***H**_ρ_* at a point in space is the result of the combined action of all magnetic charges on the magnetized material. The relationship between the magnetic field and the magnetic charges is as follows:

(1) $$H_{\rho }=\int_{V}\frac{r}{4\pi r^{3}}d\rho_{m}+\int_{S}\frac{r}{4\pi r^{3}}d\rho_{s}$$

where V and S are the volume and surface area of the ferromagnetic material, respectively. **r** is a vector pointing from the element of d*S* to P(*x*, *y*, *z*), and r is the distance between the d*S* and point P. *ρ*_m_ is the volume magnetic charge density. *ρ_s_* is the surface magnetic charge density. When the magnetic medium is uniformly magnetized, the magnetization intensity is constant, so *ρ*_m_ = −▽·***M*** = 0, meaning only surface magnetic charges exist. Otherwise, both types of magnetic charges coexist at a given point.

When d*S* is sufficiently small, the magnetization on the tiny unit area can be regarded as uniform magnetization, and the magnetic charge density d*ρ_s_* on d*S* is expressed as follows:

(2) $$d\rho_{s}=\rho_{s}dS$$

After a period of stress loading, its direction tends to be parallel to that of the excitation field. The surface magnetic charge density *ρ_s_* can be calculated using an empirical formula [35]:

(3) $$\rho_{s}=\frac{2.65}{2\pi }\left(\frac{1+h/(2a)}{1+h/(2\mu_{0}\mu_{r}a)}\right)H$$

where *H* is the applied magnetic field, corresponding to the geomagnetic field in magnetic memory testing.

The magnetic field intensity vector at an arbitrary point P near the crack from the inclined plane is resolved into components as follows:

(4) $$H_{l}=\iint \frac{2\rho_{s}e_{r_{1}}}{r_{1}^{2}}(dx_{1}dz_{1}+dy_{1}dz_{1})\;H_{r}=\iint \frac{-2\rho_{s}e_{r_{2}}}{r_{2}^{2}}(dx_{2}dz_{2}+dy_{2}dz_{2})$$

where ***e**_r_*_1_ and ***e**_r_*_2_ are the unit vectors along *r*_1_ and *r*_2_, respectively.

Since the direction of magnetic flux lines in the ferromagnetic component is parallel to the *xoz* plane, the magnetic field intensities at point P generated by the two inclined crack surfaces are as follows:

(5) $$H_{l}=\iint \frac{2\rho_{s}e_{r_{1}}}{r_{1}^{2}}dy_{1}dz_{1}=\iint \frac{2\rho_{s}e_{r_{1}}}{{\left(x_{1}-x\right)}^{2}+{\left(y_{1}-y\right)}^{2}+{\left(z_{1}-z\right)}^{2}}dy_{1}dz_{1}\;H_{r}=\iint \frac{-2\rho_{s}e_{r_{2}}}{r_{2}^{2}}dy_{2}dz_{2}=\iint \frac{-2\rho_{s}e_{r_{2}}}{{\left(x_{2}-x\right)}^{2}+{\left(y_{2}-y\right)}^{2}+{\left(z_{2}-z\right)}^{2}}dy_{2}dz_{2}$$

In summary, the resultant magnetic field intensity vector at point P is as follows:

(6) $$\begin{array}{cc} H= & H_{l}+H_{r}=\iint \frac{2\rho_{s}e_{r_{1}}}{r_{1}^{2}}dy_{1}dz_{1}-\iint \frac{2\rho_{s}e_{r_{2}}}{r_{2}^{2}}dy_{2}dz_{2}\\ = & \iint \frac{2\rho_{s}e_{r_{1}}}{{\left(x_{1}-x\right)}^{2}+{\left(y_{1}-y\right)}^{2}+{\left(z_{1}-z\right)}^{2}}dy_{1}dz_{1}\\ - & \iint \frac{-2\rho_{s}e_{r_{2}}}{{\left(x_{2}-x\right)}^{2}+{\left(y_{2}-y\right)}^{2}+{\left(z_{2}-z\right)}^{2}}dy_{2}dz_{2} \end{array}$$

The magnetic field strength at spatial detection point P near the crack can be decomposed into three directional spatial components, as shown in Figure 2c.

(7) $$H_{x}=H_{l}\cos\alpha_{1}+H_{r}\cos\alpha_{2}=H_{l}\frac{x-x_{1}}{r_{1}}+H_{r}\frac{x-x_{2}}{r_{2}}\;H_{y}=H_{l}\cos\beta_{1}-H_{r}\cos\beta_{2}=H_{l}\frac{y-y_{1}}{r_{1}}-H_{r}\frac{y-y_{2}}{r_{2}}\;H_{z}=H_{l}\cos\gamma_{1}+H_{r}\cos\gamma_{2}=H_{l}\frac{z-z_{1}}{r_{1}}+H_{r}\frac{z-z_{2}}{r_{2}}$$

As shown in Figure 2d, rotate the spatial coordinate system *oxyz* counterclockwise by an angle *θ* around the *z*-axis to obtain a new coordinate system *ox*′*y*′*z*′. The transformation relations between the old and new coordinate systems are as follows:

(8) $$x^{\prime }=x\cos\theta -y\sin\theta \;y^{\prime }=y\cos\theta +x\sin\theta \;z^{\prime }=z$$

Under the new coordinate system *ox*′*y*′*z*′, the contribution of the left inclined plane to the magnetic field strength at the detection point can be quantitatively reflected by Equation (9), and its three spatial components are given in Equation (10).

(9) $$H_{l}^{\prime }=\int_{-l}^{l}\int_{0}^{\sqrt{h^{2}+a^{2}}}\frac{2\rho_{s}e_{r1}}{{x^{\prime }}^{2}+{\left(y^{\prime }-y_{1}\right)}^{2}+{\left(z^{\prime }-z_{1}\right)}^{2}}dy_{1}dz_{1}$$

(10) $$H_{lx}^{\prime }=\int_{-l}^{l}\int_{0}^{\sqrt{h^{2}+a^{2}}}\frac{2\rho_{s}x^{\prime }}{{\left[{x^{\prime }}^{2}+{\left(y^{\prime }-y_{1}\right)}^{2}+{\left(z^{\prime }-z_{1}\right)}^{2}\right]}^{3/2}}dy_{1}dz_{1}\;H_{ly}^{\prime }=\int_{-l}^{l}\int_{0}^{\sqrt{h^{2}+a^{2}}}\frac{2\rho_{s}(y^{\prime }-y_{1})}{{\left[{x^{\prime }}^{2}+{\left(y^{\prime }-y_{1}\right)}^{2}+{\left(z^{\prime }-z_{1}\right)}^{2}\right]}^{3/2}}dy_{1}dz_{1}\;H_{lz}^{\prime }=\int_{-l}^{l}\int_{0}^{\sqrt{h^{2}+a^{2}}}\frac{2\rho_{s}(z^{\prime }-z_{1})}{{\left[{x^{\prime }}^{2}+{\left(y^{\prime }-y_{1}\right)}^{2}+{\left(z^{\prime }z_{1}\right)}^{2}\right]}^{3/2}}dy_{1}dz_{1}$$

Rotate the coordinate system *oxyz* clockwise by an angle *θ* around the oz axis to obtain a new coordinate system *ox*″*y*″*z*″. The transformation relationship between the original and new coordinate systems is given in Equation (11).

(11) $$x^{\prime \prime }=x\cos(-\theta )-y\sin(-\theta )=x\cos\theta +y\sin\theta \;y^{\prime \prime }=y\cos(-\theta )+x\sin(-\theta )=y\cos\theta -x\sin\theta \;z^{\prime \prime }=z$$

In the new coordinate system *ox*″*y*″*z*″, the contribution of the right inclined plane to the magnetic field intensity at the detection point can be quantitatively reflected by Equation (12), and its three spatial components are expressed in Equation (13).

(12) $$H_{r}^{\prime \prime }=\int_{-l}^{l}\int_{0}^{\sqrt{h^{2}+a^{2}}}\frac{-2\rho_{s}e_{r2}}{{\left[{x^{\prime \prime }}^{2}+{\left(y^{\prime \prime }-y_{2}\right)}^{2}+{\left(z^{\prime \prime }-z_{2}\right)}^{2}\right]}^{2}}dy_{2}dz_{2}$$

(13) $$H_{rx}^{\prime \prime }=\int_{-l}^{l}\int_{0}^{\sqrt{h^{2}+a^{2}}}\frac{-2\rho_{s}x^{\prime \prime }}{{\left[{x^{\prime \prime }}^{2}+{\left(y^{\prime \prime }-y_{2}\right)}^{2}+{\left(z^{\prime \prime }-z_{2}\right)}^{2}\right]}^{3/2}}dy_{2}dz_{2}\;H_{ry}^{\prime \prime }=\int_{-l}^{l}\int_{0}^{\sqrt{h^{2}+a^{2}}}\frac{-2\rho_{s}(y^{\prime \prime }-y_{2})}{{\left[{x^{\prime \prime }}^{2}+{\left(y^{\prime \prime }-y_{2}\right)}^{2}+{\left(z^{\prime \prime }-z_{2}\right)}^{2}\right]}^{3/2}}dy_{2}dz_{2}\;H_{rz}^{\prime \prime }=\int_{-l}^{l}\int_{0}^{\sqrt{h^{2}+a^{2}}}\frac{-2\rho_{s}(z^{\prime \prime }-z_{2})}{{\left[{x^{\prime \prime }}^{2}+{\left(y^{\prime \prime }-y_{2}\right)}^{2}+{\left(z^{\prime \prime }-z_{2}\right)}^{2}\right]}^{3/2}}dy_{2}dz_{2}$$

The three spatial components of the magnetic field intensity at the detection point, decomposed along the coordinate axes and derived from the above theory, are expressed in Equation (14), which can be quantitatively solved by combining Equations (10) and (13).

(14) $$H_{x}=H_{lx}^{\prime }+H_{rx}^{\prime \prime }\;H_{y}=H_{ly}^{\prime }+H_{ry}^{\prime \prime }\;H_{z}=H_{lz}^{\prime }+H_{rz}^{\prime \prime }$$

The proposed magnetic dipole decomposition modeling method is developed for the inclined surface structure of V-shaped cracks by projecting the magnetic charges on inclined surfaces onto coordinate planes, which effectively addresses the difficulty of conventional models in handling inclined cracks. This approach enables the decoupled calculation of magnetic signal components (*H_x_*/*H_y_*), significantly reducing the computational complexity of traditional magnetic dipole models and improving engineering applicability. Furthermore, the model is applied to characterize the full-cycle evolution of cracks from micrometer-scale to macroscopic scale, extending the limitation of available models that are mostly applicable to macroscopic stationary cracks.

## 3. Results

To verify the correctness of the proposed theoretical model, three-point bending experiments and finite element simulation are carried out on Q345 steel specimens with prefabricated V-shaped cracks. The test scheme, data acquisition and result analysis are presented in this section.

### 3.1. Test Specimens and Materials

**Figure 3:**
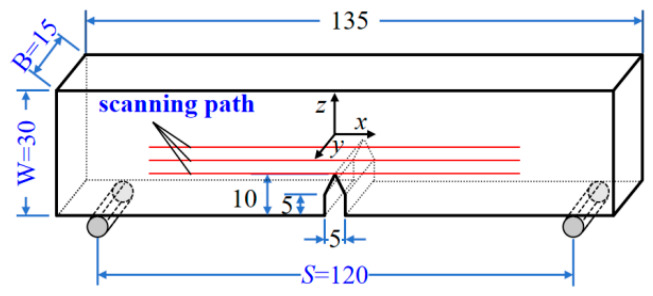
Specimen (Unit: mm).

Defect specimens were machined into standard three-point bending specimens in accordance with ISO 12135:2021 Metallic materials-Unified test method for quasi-static fracture toughness, satisfying S/W = 4 and W/B = 2. The overall dimensions of the specimen are 135 mm × 30 mm × 15 mm. A V-notch defect was processed from one side of the specimen, and the detailed dimensions are shown in Figure 3. Regarding the surface roughness: the surface in contact with the bending indenter is Ra0.8, and the two detection surfaces corresponding to the V-notch are Ra1.6. To subsequently collect displacement and strain information during the test, the notch region of the specimen was polished with sandpaper, and a speckle pattern was sprayed onto one side of the scanning plane for digital image correlation.

The specimen material is Q345 steel, which is similar to the American steel grade ASTM A709 Grade 50. This steel contains low contents of sulfur (S) and phosphorus (P), making it less prone to brittle fracture. The chemical composition and mechanical properties of the specimen material are listed in Table 1 and Table 2, respectively.

### 3.2. Test Method and Procedure

A microscopic observation platform was used to monitor, measure and analyze the crack tip in the stress concentration region in real time. On the basis of in situ observation of the specimen under varying loads, the surface magnetic field signals corresponding to the crack size were recorded.

**Figure 4:**
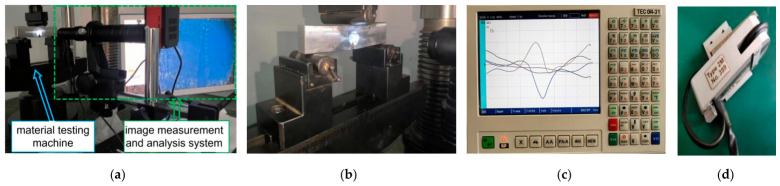
Experimental equipment: (**a**) In situ microscopic imaging system; (**b**) Three-point bending experiment; (**c**) MMM testing instrument; (**d**) Detection probe.

Three parallel evenly spaced scanning lines were set on the specimen surface, and the detection plane was perpendicular to the north–south direction. The spacing between detection lines for the V-notch defect specimen was 5 mm. The V-notch defect specimen was loaded in a three-point bending configuration, as illustrated in Figure 4. Mechanical tests were performed on an MTS810 material testing machine (Changchun, China) with a maximum load capacity of 250 kN. A TSC-5M-32 MMM detector, manufactured by Energodiagnostika Co., Ltd. (Moscow, Russia), was utilized to collect MMM signals. For crack length measurement and observation, the HG-4008T image measurement and analysis system was employed. This system can accurately capture and clearly display crack images; through image analysis algorithms, it identifies the crack profile from the captured images and then converts the profile into the actual crack length.

**Figure 5:**
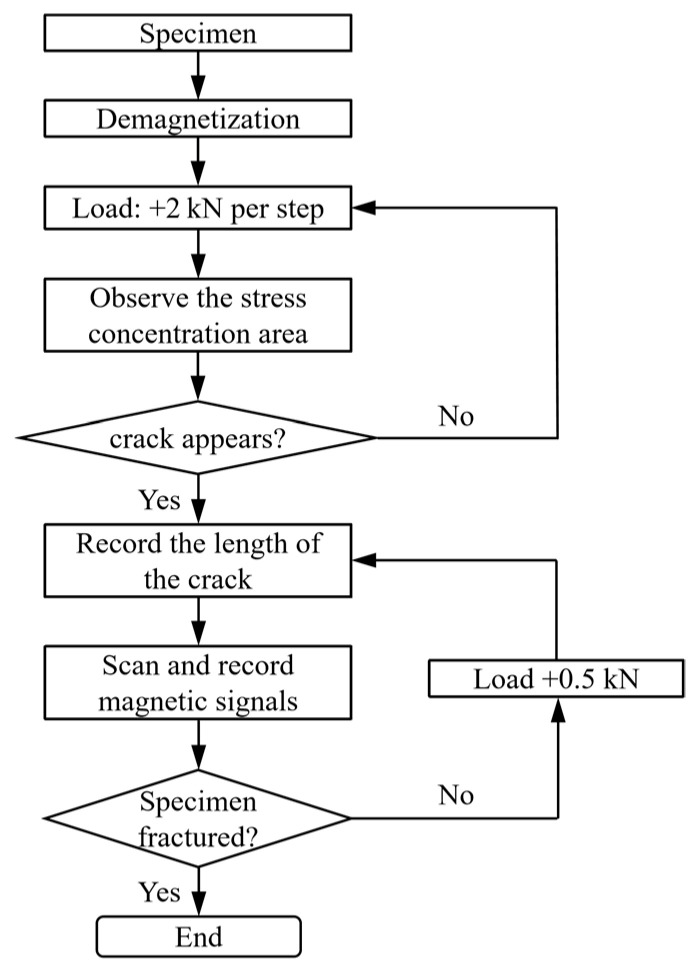
Process diagram for crack detection.

The actual flow of crack scanning is shown in Figure 5. The detailed test procedures are as follows: Firstly, the prepared specimen was demagnetized to eliminate the influence of the material’s self-leakage magnetic field. Then, the initial magnetic memory signals of each cracked specimen were detected and stored. The specimen was loaded according to the predetermined load, and a CCD industrial camera was used to observe potential crack initiation sites in the defect region. Once a crack appeared, the crack length was recorded, and scanning was performed sequentially along each detection line. After crack initiation, the specimen was loaded step-by-step with a load increment of 0.5 kN (corresponding to a nominal stress of 4 MPa) until fracture occurred.

Various inevitable factors contribute to the dispersion of experimental results, including geometric deviations arising from specimen machining and V-shaped notch prefabrication, detection errors induced by sensor lift-off height and ambient geomagnetic interference, synchronization deviations in the loading process and reading errors of the image measurement system, as well as property scatter caused by the inhomogeneous local microstructure of materials. For this reason, repeated tests were conducted on five groups of parallel specimens under identical experimental conditions. The results demonstrate that the crack initiation load, critical crack length, and peak-valley characteristics and evolution trends of magnetic signals all exhibit excellent consistency and reliable repeatability among different specimens.

### 3.3. Test Results and Model Validation

**Figure 6:**
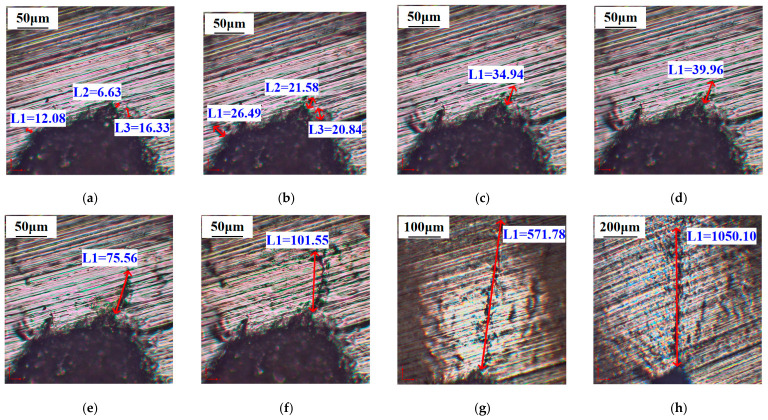
Microscopic morphology of surface cracks under different applied loads: (**a**) 24 kN; (**b**) 26 kN; (**c**) 30 kN; (**d**) 40 kN; (**e**) 45 kN; (**f**) 47.5 kN; (**g**) 50 kN; (**h**) 52 kN.

Observation on the tip of the prefabricated V-notch via microscopy reveals that the signal waveform distorts when the load increases to 22 kN, whereas no visible cracks are observed under the microscope. As the load increases from 24 kN to 26 kN, several microcracks appear in the signal distortion region, and each crack exhibits a similar growth rate, as shown in Figure 6a,b. When the load ranges from 30 kN to 45 kN, the growth rate of some cracks gradually slows down, and a dominant crack forms after competition among these cracks, as illustrated in Figure 6c–e. After the load increases to 47.5 kN, the dominant crack propagates rapidly, as presented in Figure 6f. At a load of 50 kN, the length of the dominant crack extends to 571.78 μm, as shown in Figure 6g. When the load increases to 52 kN, the length of the dominant crack exceeds 1 mm, as displayed in Figure 6h.

**Figure 7:**
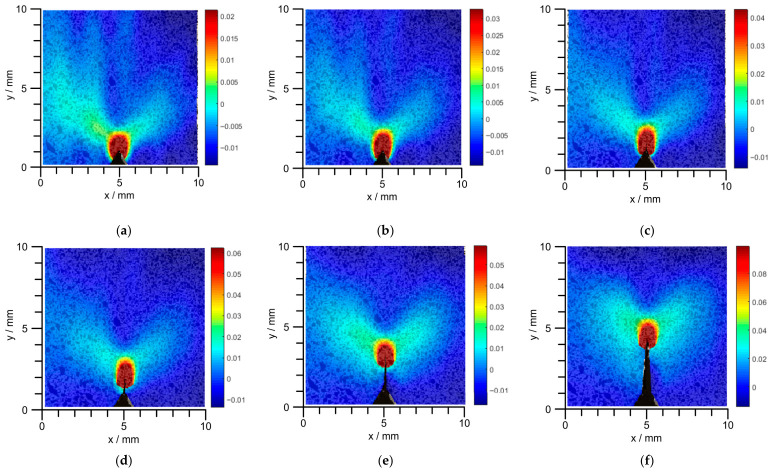
Strain field contour cloud in the direction of crack opening under external loading; (**a**) 50 kN; (**b**) 51 kN; (**c**) 51.5 kN; (**d**) 52 kN; (**e**) 52.5 kN; (**f**) 53 kN.

**Figure 8:**
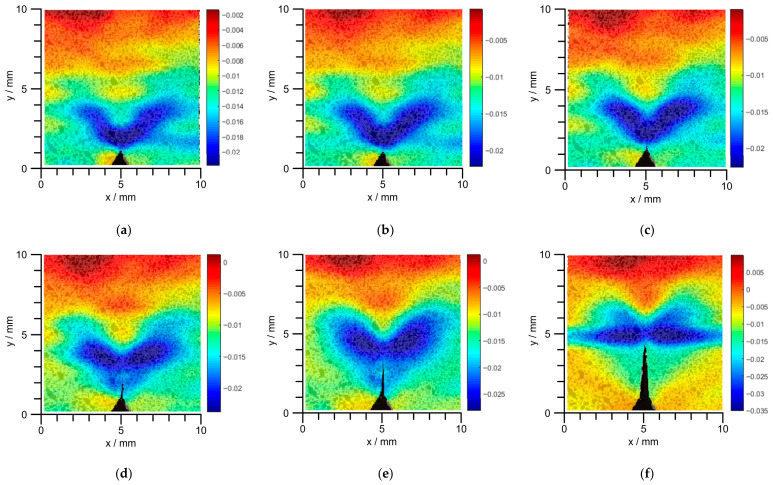
Strain field contour cloud in the direction of load application under external loading; (**a**) 50 kN; (**b**) 51 kN; (**c**) 51.5 kN; (**d**) 52 kN; (**e**) 52.5 kN; (**f**) 53 kN.

Macroscopic crack propagation can be identified by the Digital Image Correlation (DIC) technique, which also enables the recording of the strain field in the crack region. The evolution of the strain field along the crack opening direction is presented in Figure 7. During crack development, the strain magnitude at the crack tip is distinctly higher than that in other regions. The strain field along the loading direction is shown in Figure 8, exhibiting a wider strain-affected zone. With the growth of the crack, the strain at the initial crack opening position gradually becomes consistent with that in the non-stress-concentrated region. This regularity can be conveniently utilized to identify the location of the crack tip.

**Figure 9:**
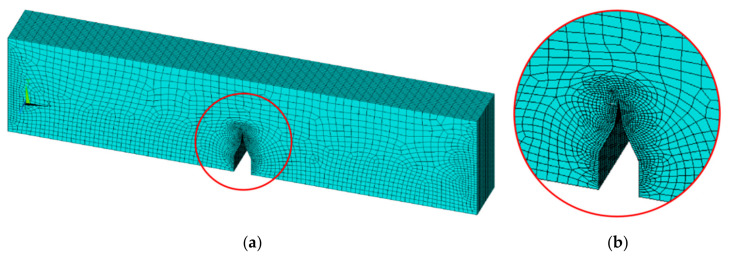
Finite Element Mesh Division of Cracks: (**a**) crack-containing model; (**b**) enlarged view.

To verify the consistency between the theoretical model and experimental results, a combination of finite element simulation and numerical calculation was employed for comparison with the experimental data. First, for the planar V-notch specimen, the finite element method was used to analyze the standard specimen with a prefabricated crack. The established finite element model is shown in Figure 9. To introduce the crack, a crack was directly created in the geometric model, and finer mesh refinement was applied near the crack tip, as shown in Figure 9b. In the finite element model, a scanning path perpendicular to the crack plane was set along the crack propagation direction, with the crack tip as the origin, extending 20 mm to both sides to cover the plastic zone boundary. The magnetic field intensity components (*H_x_*, *H_y_*) at the nodes along this path were extracted through the post-processing module.

Simulation results are limited by the assumptions of the theoretical model, while uncontrollable factors in practice lead to differences between the experimental magnetic field data on the specimen surface and the simulation or model prediction results in both trend and magnitude. Nevertheless, simulation can still effectively reveal the magnetic field variation law under specific conditions.

**Figure 10:**
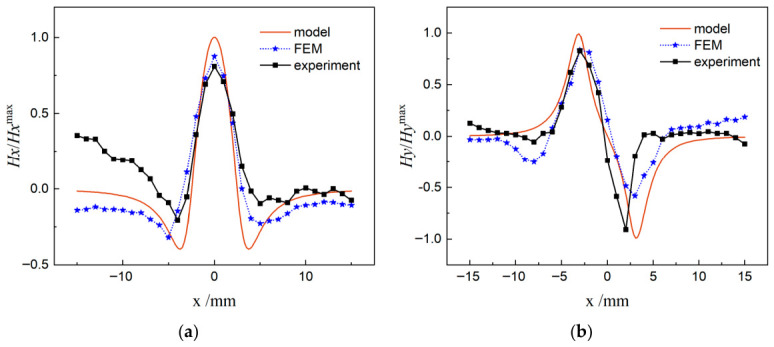
Model validation (prefabricated cracks); (**a**) *H_x_*; (**b**) *H_y_*.

To verify the validity of the theoretical model, a normalization method was adopted to compare and analyze the magnetic field data from simulation, experiment and model calculation, with the results shown in Figure 10. It is found that the theoretical model is in good agreement with the finite element simulation and experimental signals in the region near the crack tip, and the agreement is better than that in regions far from the crack.

Based on the simulation and experimental data for the V-shaped crack, the model accuracy was quantified using Root-Mean-Square Error (RMSE) and Relative Error. Within the 20 mm range near the crack tip, the average errors of the magnetic field components are:

V-shaped crack specimen:

Relative error of the tangential component = 15.2% (RMSE = 0.83 A/m),

Relative error of the normal component = 17.6% (RMSE = 0.98 A/m).

In summary, the three-point bending tests and finite element simulations on specimens with prefabricated planar cracks show that both the crack evolution characterization model established in this study and the finite element results are consistent with the experimental data. Meanwhile, the signal characteristics of the pre-cracked specimen are obvious: the single-peak feature of *H_x_* and the peak-to-peak feature of *H_y_* are distinct.

Under the same conditions, the simulation results of the proposed model and the classical magnetic dipole model are compared in the following Table 3.

The model proposed in this paper features lower computational complexity and stronger engineering applicability while achieving comparable accuracy.

The errors are mainly attributed to model idealization and nonlinear simplification, material magnetization history, localized plastic heterogeneity and magnetic domain dispersion, as well as experimental factors including sensor lift-off, environmental magnetic interference, surface roughness and loading synchronization deviation, together with numerical errors caused by mesh generation, integral approximation and coordinate transformation.

## 4. Discussion

Based on the test and simulation results, the evolutionary laws of crack length and magnetic memory signals under external load are further discussed in this section.

### 4.1. Evolution of Crack Length with Applied Load

**Figure 11:**
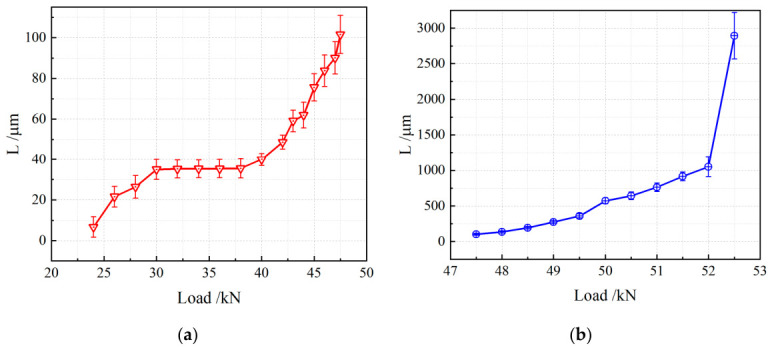
Relationship between crack length and load: (**a**) early stage; (**b**) later stage.

The variation in crack length with applied load is shown in Figure 11. Figure 11a presents the evolution of crack length with load at the microscale, within the range of crack lengths below 100 μm. At this scale, crack length exhibits three distinct stages as load increases.

Stage 1: At the initial detectable stage of cracking, crack length *L* increases gradually with rising load. This indicates that internal defects in the material begin to propagate under loading, and cracks initiate and develop progressively.

Stage 2: When the crack length extends to 30–40 μm with increasing load, numerous microcracks undergo random competition. At this stage, the internal stress state of the material balances crack propagation, and the crack remains relatively stable within this size range without significant growth.

Stage 3: With a further increase in load, the equilibrium is broken, and crack length resumes steady growth. This stage indicates that the continuous load increase causes the internal stress to exceed the critical value for crack stability, driving further crack propagation.

As observed in Figure 11b, when the crack length exceeds 100 μm, crack growth remains relatively steady over most of the loading range. However, as the material approaches its critical failure state, the crack length increases abruptly. This demonstrates that the crack propagation rate rises sharply just before fracture, leading to a rapid loss of structural integrity.

**Figure 12:**
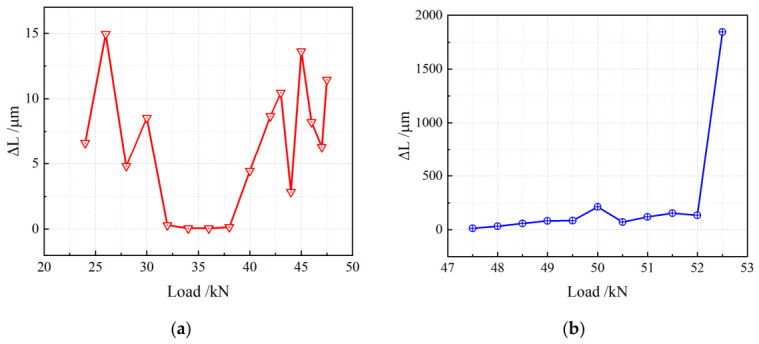
Relationship between crack growth increment and load: (**a**) early stage; (**b**) later stage.

To quantitatively investigate the relationship between crack growth increment Δ*L* and load, further data analysis was conducted, with results presented in Figure 12. A clear stage-dependent behavior of crack growth increment Δ*L* versus load can be observed.

At the initial stage, Δ*L* increases rapidly with increasing load, indicating a sensitive response of cracking to loading. The crack growth increment then enters a plateau stage, where it remains nearly constant despite the increasing load, suggesting that an internal mechanism restrains further crack growth temporarily. Subsequently, when the load exceeds a certain threshold, Δ*L* enters a stable growth stage, showing an approximately linear relationship with load, meaning that the increasing load continuously drives crack propagation at a relatively steady rate. Finally, immediately before fracture, the crack growth increment rises steeply, indicating an accelerated crack propagation rate and rapid degradation of structural integrity.

**Figure 13:**
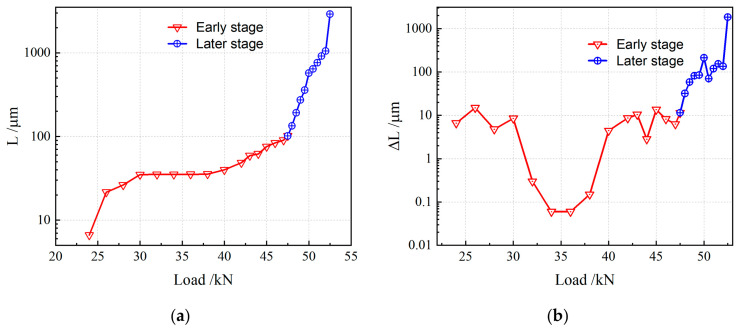
Relationship between crack total variation and load: (**a**) Crack length; (**b**) Crack growth increment.

Based on the above analysis, crack evolution with load exhibits distinct stages from a global perspective, as illustrated in Figure 13. The overall trends of crack length and crack growth increment with increasing load are summarized as follows.

In Figure 13a, the crack length increases significantly at the early stage with rising load, showing a large growth rate. This indicates that load strongly drives crack extension, and the crack propagates rapidly inside the material. As load increases further, the crack growth rate gradually stabilizes, implying that the crack propagation is influenced by internal microstructural factors and enters a steady-growth phase.

Figure 13b shows the relationship between Δ*L* and load. Δ*L* fluctuates within a certain range at the early stage, reflecting the uncertainty and complexity of the crack propagation process. After the load reaches a threshold value, Δ*L* changes noticeably and rises sharply in the later stage, indicating that the material is approaching critical failure with a significantly accelerated crack growth rate.

### 4.2. Evolution of Magnetic Signals for Prefabricated Cracks

**Figure 14:**
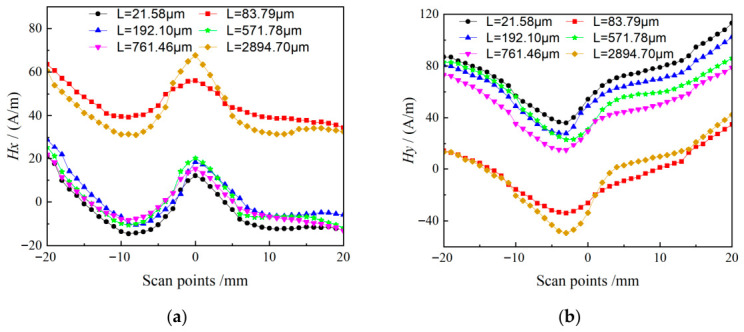
MMM Signal waveforms of different cracks: (**a**) *H_x_*; (**b**) *H_y_*.

Through observation and analysis of the curves in Figure 14, most signal waveforms show similar trends, and local characteristic peaks or valleys appear at the scanning positions corresponding to the crack tip.

During crack development, the signal waveforms mostly cluster together with small overall shifts, indicating that the magnetic memory signals are relatively stable. However, significant jumps in the overall waveforms occur at crack lengths of 83.79 μm and 2894.7 μm, corresponding to the transitions from microcrack initiation to mesoscale short crack propagation, and from mesoscale short cracks to macrocrack propagation, respectively.

From the viewpoint of magnetism and materials science, magnetic signals reflect the internal energy state of the material. The abnormal waveform changes at these two characteristic crack lengths imply that the internal energy of the material undergoes qualitative changes, breaking the original stable energy state. Such energy variations are likely closely related to crack geometry, propagation mechanisms, and microstructural evolution, and deserve further investigation.

**Figure 15:**
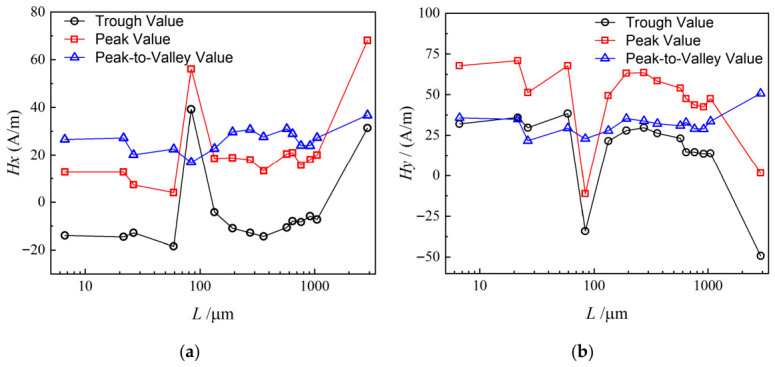
Relationship between MMM characteristics and crack length: (**a**) *H_x_*; (**b**) *H_y_*.

Figure 15 shows the relationship between magnetic signal characteristic values and crack length over the entire crack propagation cycle. Specifically, the peak and valley values (representing signal position) change noticeably near crack lengths of 100 μm and 3000 μm. At these two critical lengths, the values and trends of the peaks and valleys deviate significantly from those at other lengths, reflecting abrupt changes in the material’s internal magnetic properties.

When the crack length is approximately 100 μm, K_I_ = 53.11 MPa·m^1/2^ and J_e_ = 12.46 kJ/m^2^. The specimen is on the verge of crack initiation. If the fracture toughness of the tested material is relatively low (e.g., 50~55 MPa·m^1/2^), crack initiation may have already occurred. The plastic zone at the crack tip expands, and the crack is about to enter stable propagation, i.e., subcritical propagation.

When the crack length reaches approximately 3000 μm, K_I_ = 84.75 MPa·m^1/2^ and J_e_ = 31.72 kJ/m^2^. The specimen reaches the critical fracture state, and the crack will inevitably undergo unstable propagation. At this moment, the plastic zone at the crack tip is extremely large, and linear elastic fracture mechanics is no longer applicable. Nevertheless, the calculated parameters exceed the fracture resistance of the material, indicating that fracture has already taken place. In the experiment, this state corresponds to the descending stage after the load reaches its peak, or unstable fracture following the pop-in phenomenon.

When the crack length is less than 100 μm, crack evolution is dominated by dislocation multiplication and microvoid initiation, accompanied by a relatively weak magnetic perturbation. As the crack extends to around 100 μm, the constraint effect of grain boundaries gradually fails, and the crack propagates in a transgranular manner, forcing the reconstruction of magnetic domain structures and inducing the first characteristic jump of the magnetic signal. When the crack length approaches approximately 3000 μm, the plastic zone penetrates the cross-section of the component, and the crack enters the unstable propagation stage. The local magnetic permeability of the material changes drastically, leading to an obvious rise and a second characteristic jump of the magnetic signal.

The peak-to-peak value, which characterizes the magnitude of signal variation, behaves differently. It increases substantially only when the material is near fracture. This indicates that the signal variation is relatively stable during most of the propagation process, but the fluctuation amplitude increases significantly at the critical failure stage.

These features are fully reflected in the waveform evolution at different crack lengths, including the signal divergence from the clustered region shown in Figure 14. The signal divergence is mainly exhibited as obvious shifts in peak and valley positions at specific crack lengths. In contrast, signal distortion becomes prominent only near final fracture, reflecting the strong influence of drastic microstructural changes on the magnetic signal at the critical failure state.

A complex but regular relationship exists between crack length and magnetic signal characteristics. These findings provide valuable references for monitoring crack propagation using magnetic signals.

**Figure 16:**
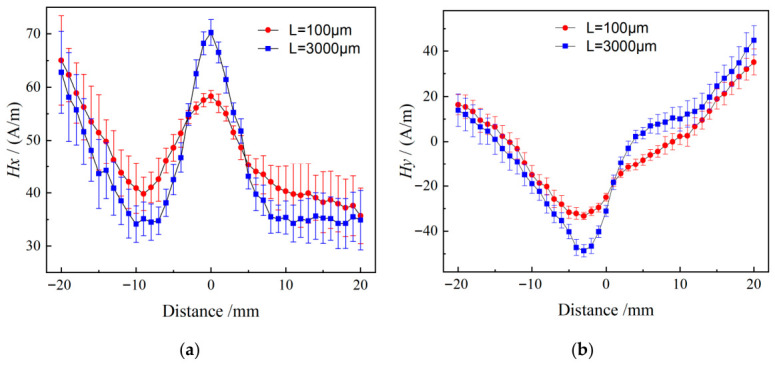
Relationship between signal error and detection distance in the critical interval: (**a**) *H_x_*; (**b**) *H_y_*.

Investigations on the evolution of MMM signal error with the distance between the measuring point and the crack tip show that the signal error is mainly concentrated in the far-field detection region, while the optimal consistency between theoretical predictions and experimental results is achieved in the near-tip region (as shown in Figure 16). Compared with far-field positions, the magnetic memory signal near the crack tip is dominated by the crack singular field, which significantly suppresses the deviation between numerical calculations and measured data.

This study takes revealing the intrinsic magneto-mechanical coupling law during the full-life-cycle evolution of V-shaped cracks as the core research objective. Several concise and intuitive characteristic parameters, including peak and valley positions as well as signal amplitude, are selected for analysis. These parameters possess clear physical connotation, high extraction stability and excellent practicability in engineering scenarios. They can effectively weaken the shielding effect of complex algorithms on internal evolution mechanisms, highlight the essential correlation between magnetic signal response and crack propagation behavior, and ensure the research focuses on the interpretation of underlying physical mechanisms.

## 5. Conclusions

(1) A magnetic dipole model and its decomposed field formulation are established for V-shaped cracks, effectively reducing computational complexity and improving the engineering applicability of MMM-based crack characterization.
(2) Synchronized in situ three-point bending tests demonstrate that MMM can predict crack nucleation before visible initiation and identify multi-scale cracks from microcracks to macroscopic cracks.
(3) Crack evolution under external load exhibits distinct stages: initial slow growth, short crack competition at 30~40 μm, steady propagation, and abrupt acceleration before fracture. MMM signal peaks and valleys mutate at critical crack lengths, corresponding to material internal energy state transitions.
(4) The proposed theoretical model agrees well with finite element simulation and experimental data near the crack tip, with relative errors of tangential and normal components both below 18%, validating model accuracy and reliability.
(5) This work realizes full-cycle magnetic signal characterization from microcracks to macrocracks and provides a theoretical and experimental foundation for early damage detection, crack propagation monitoring, and service life evaluation of ferromagnetic engineering structures.

The critical crack lengths and magnetic memory signal calibration relationships derived in this work are established based on Q345 steel specimens with a single symmetric V-shaped notch under quasi-static three-point bending loading.

In the present modeling framework, uniform magnetic charge distribution and idealized V-shaped crack geometry are adopted, whereby reasonable simplifications are made for rough crack surfaces and asymmetric crack configurations. However, the coupled influences of initial magnetization history, multiaxial stress state and ambient temperature are not taken into account in the current model. Furthermore, the numerical accuracy in the far-field region manifests a minor discrepancy relative to the vicinity of the crack tip.

Further extending the proposed model and characteristic laws to other steel grades, diverse crack types (e.g., surface cracks, embedded cracks, and mixed-mode cracks), and complex loading histories such as dynamic fatigue and impact loading, can serve as a significant research direction for the quantitative characterization of MMM in future work.

The full-cycle characterization framework established in this study can be further integrated with multi-dimensional feature sets, quantitative inversion algorithms and machine learning classification methods, so as to improve the detection accuracy and intelligent recognition capability under multiple working conditions, various defect forms and different material systems. The revealed critical transition law of crack evolution and signal characteristics can provide physical prior knowledge and theoretical support for the above intelligent detection methods.

## Figures and Tables

**Table 1:** Chemical Composition of Experimental Material (wt.%).

C	Si	Mn	Cr	P	S	Ni	Cu
0.12~0.20	0.20~0.60	1.20~1.60	≤0.30	≤0.035	≤0.035	≤0.30	≤0.25

**Table 2:** Mechanical Properties of Experimental Material.

Elastic Modulus (GPa)	Yield Strength (MPa)	Ultimate Strength (MPa)	Elongation Rate (%)	Poisson Ratio
201	345	450–600	19	0.26

**Table 3:** Quantitative Comparison Between the Proposed Model and Mainstream Magnetic Dipole Models.

Model	Tangential Component Error/%	Normal Component Error/%	RMSE/(A/m)	Calculation Time/s	Advantages
Traditional Linear Magnetic Dipole Model [7]	24.7	26.3	1.36	12.8	Simple formula but low accuracy
Rectangular Magnetic Dipole Model [36]	18.5	20.1	1.12	18.5	Improved accuracy but higher time consumption
Magnetoelastic Coupling Magnetic Dipole Model [12]	16.8	19.2	1.05	22.3	High accuracy but high complexity
Proposed Decomposed Magnetic Dipole Model for V-Shaped Cracks	15.2	17.6	0.98	6.7	Highest accuracy, shortest time consumption, and strong engineering applicability

## Data Availability

The original contributions presented in this study are included in the article. Further inquiries can be directed to the corresponding author.

## References

[1] Wang D., Wang J., Yao K. (2026). Magnetomechanical model for contact damage based on magnetic memory. Int. J. Mech. Sci..

[2] Wieczorska A., Kosoń-Schab A. (2023). Using Metal Magnetic Memory to Evaluate the Effect of Welding Method and Weld Temperature on Magnetic Field Strength in Structural Steel. Materials.

[3] Jiang H., Zhang L., Fan J., Zhang Z., Wang K. (2025). A study on fatigue life evaluation of 42CrMo steel under cyclic loading based on metal magnetic memory method. NDTE Int..

[4] Zuo F., Dong M., Xu G., Ge H., Su S., Wang W., Wen Y., Wu Z. (2026). Research on hidden damage in orthotropic steel bridge deck diaphragms based on metal magnetic memory technology. Structures.

[5] Li X., Sheng G., Meng Z., Qin F., Liu Z. (2023). Magnetic Charge Model for Leakage Signals from Surface Defects in Ferromagnetic Material. Materials.

[6] Wang H., Xu Z., Cai D., Dong L., Ma G., Wang H., Liu B. (2022). Stress Evaluation of Welded Joints with Metal Magnetic Memory Testing Based on Tension–Compression Fatigue Test. Materials.

[7] Leng J.C., Xing H.Y., Zhou G.Q., Gao Y.T. (2013). Dipole modelling of metal magnetic memory for V-notched plates. Insight.

[8] Liu B., Fu P., Li R., He P., Dong S. (2019). Influence of Crack Size on Stress Evaluation of Ferromagnetic Low Alloy Steel with Metal Magnetic Memory Technology. Materials.

[9] Jung G., Seo J., Park Y. (2019). Comparison between Magnetic Field Distribution Analysis and Metal Magnetic Memory (MMM) Testing Results Around Artificial Cracks under Loads. J. Magn..

[10] Oca-Mora N.J.M.D., Woo-García R.M., Sánchez-Vidal A., Galván-Martínez R., Orozco-Cruz R., Carmona-Hernández A., Herrera-May A.L., Restrepo J., Algredo-Badillo I., López-Huerta F. (2023). Simulation and Detection of Rectangular Magnetic Cracks in Metallic Plates. J. Nondestruct. Eval..

[11] Liu L., Bao S. (2024). Experimental Research on the Effect of Cracks on Metal Magnetic Memory Signals. Eng. Mech..

[12] Li X., Liao K., He G., Zhao J. (2024). Research on magnetoelastic coupling model based on magnetic dipole theory. Insight.

[13] Huang H., Jiang S., Wang Y., Zhang L., Liu Z. (2014). Characterization of spontaneous magnetic signals induced by cyclic tensile stress in crack propagation stage. J. Magn. Magn. Mater..

[14] Chongchong L., Lihong D., Haidou W., Guolu L., Binshi X. (2016). Metal magnetic memory technique used to predict the fatigue crack propagation behavior of 0.45%C steel. J. Magn. Magn. Mater..

[15] Hu Z., Fan J., Wu S., Dai H., Liu S. (2018). Characteristics of Metal Magnetic Memory Testing of 35CrMo Steel during Fatigue Loading. Metals.

[16] Zhou W., Fan J., Ni J., Liu S. (2019). Variation of Magnetic Memory Signals in Fatigue Crack Initiation and Propagation Behavior. Metals.

[17] Li J., Su S., Wang W. (2025). Research on fatigue crack propagation behavior and reliability evaluation of steel bridge. J. Constr. Steel Res..

[18] Ye J., Guo Z., Zeng S., Xu M. (2024). Magnetic memory testing towards fatigue crack propagation of Q235 steel for remanufacturing. Int. J. Appl. Electrom..

[19] Ni C., Hua L., Wang X. (2018). Crack propagation analysis and fatigue life prediction for structural alloy steel based on metal magnetic memory testing. J. Magn. Magn. Mater..

[20] Xu K., Yang K., Liu J., Chen X., Wang Y. (2020). Investigation of Magnetic Memory Signal of Propagation of Buried Crack under Applied Load. Res. Nondestruct. Eval..

[21] Lee J., Wang D., Dharmawan I.D.M.O. (2022). Theoretical Model of Self-Magnetic Flux Leakage and Its Application in Estimating the Depth Direction of a Fatigue Crack. Appl. Sci..

[22] Bao S., Li Y. (2026). Evaluation of cracks in wire and arc additive manufacturing components using magnetic memory technology. Measurement.

[23] Liu B., Feng G., He L., Luo N., Ren J., Yang L. (2021). Quantitative Study of MMM Signal Features for Internal Weld Crack Detection in Long-Distance Oil and Gas Pipelines. IEEE Trans. Instrum. Meas..

[24] Su S., Li J., Wang W., Liu X., Zuo F., Deng R. (2024). Metal magnetic memory characterization of fatigue crack propagation of Q345qD bridge steel under the influence of stress ratio. J. Magn. Magn. Mater..

[25] Su S., Yang Y., Wang W., Ma X. (2021). Crack propagation characterization and statistical evaluation of fatigue life for locally corroded bridge steel based on metal magnetic memory method. J. Magn. Magn. Mater..

[26] Sanqing S., Junting L., Wei W., Xinwei L., Fuliang Z., Ruize D. (2024). Study of fatigue crack growth behavior and reliability update analysis of steel bridges. Chin. Civ. Eng..

[27] Shijie D., Hailong C., Wanfeng G., Zhaoyi J., Xiaotao Z. (2024). Research on defect image inversion method based on multiple lift-off values magnetic memory signals. Meas. Control..

[28] Li J., Zhang N., Zhang Y. (2026). Fatigue damage detection and assessment of standard plate specimens via metal magnetic memory testing. NDTE Int..

[29] Chen L., MingJiang S., Liang Y. (2026). A data augmentation method for pipeline weld defects based on LSTM-SSL-ACGAN. Nondestruct. Test. Eval..

[30] Shi P., Jin K., Zhang P., Xie S., Chen Z., Zheng X. (2018). Quantitative Inversion of Stress and Crack in Ferromagnetic Materials Based on Metal Magnetic Memory Method. IEEE Trans. Magn..

[31] Xin J., Li R., Chen J., Lu R., Liu C., Su Z., He R., Zhu H. (2023). A crack characterization model for subsea pipeline based on spatial magnetic signals features. Ocean. Eng..

[32] Guo P., Chen T., Lian X., Ye J., Guan W., Chen X. (2017). Detection of Cracks in 25Cr35NiNb Ethylene Pyrolysis Furnace Tubes by Metal Magnetic Memory Technique. J. Press. Vessel. Technol..

[33] Dubov A., Dubov A., Kolokolnikov S., Marchenkov A. (2025). Detection of hot water boiler tubes inner surface damages at early stage of their development using the metal magnetic memory method. Weld. World.

[34] Villegas-Saucillo J.J., Díaz-Carmona J.J., Escarola-Rosas M.A., Vázquez-Leal H., Martínez-Castillo J., Herrera-May A.L. (2021). Measurements of the Magnetic Field Variations Related with the Size of V-Shaped Notches in Steel Pipes. Appl. Sci..

[35] Hao G., Li X., Yu J., Xu H., Bu L., Luo W., Guo J. (2024). High Lift Value Metal Pipeline Detection Model Based on Metal Magnetic Memory 3-D Differential Method. IEEE Sens. J..

[36] Villegas-Saucillo J.J., Díaz-Carmona J.J., Cerón-Álvarez C.A., Juárez-Aguirre R., Domínguez-Nicolás S.M., López-Huerta F., Herrera-May A.L. (2019). Measurement System of Metal Magnetic Memory Method Signals around Rectangular Defects of a Ferromagnetic Pipe. Appl. Sci..

